# Management of Priapism: Results of a Nationwide Survey and Comparison with International Guidelines

**DOI:** 10.5152/tud.2023.22209

**Published:** 2023-07-01

**Authors:** Arif Kalkanli, Salih Zeki Sönmez, Mine Guvel, Erdogan Aglamis, Seyhmuz Araz, Ahmet Asfuroglu, Huseyin Kursad Avci, Memduh Aydin, Murat Aydos, Ugur Balci, Caner Baran, Yavuz Bastug, Numan Baydilli, Omer Bayrak, Can Benlioglu, Ibrahim Halil Bozkurt, Kerem Bursali, Utku Can, Seref Coser, Mehmet Caglar Cakici, Gokhan Calik, Ali Cift, Nusret Can Cilesiz, Demirhan Orsan Demir, Murat Demir, Huseyin Cihan Demirel, Murat Dursun, Erhan Demirelli, Berk Yasin Ekenci, Mithat Eksi, Giray Ergin, Ismail Emre Ergin, Anil Erkan, Onur Fikri, Cem Tugrul Gezmis, Abdullah Gül, Muhammet Guzelsoy, Muhammed Arif Ibis, Abdurrahman Inkaya, Tumay Ipekci, Ahmet Karakeci, Kadir Karkin, Coskun Kaya, Ozgur Kazan, Mustafa Koray Kirdag, Yigit Cagri Kizilcay, Burak Koseoglu, Emrah Kucuk, Serkan Gonultas, Mehmet Sezai Ogras, Ahmet Olgun, Eser Ordek, Isa Ozbey, Mehmet Sarier, Samet Senel, Ahmet Tahra, Tuncay Toprak, Mehmet Yigit Yalcin, Abdullah Hizir Yavuzsan, Selim Yazar, İbrahim Hacıbey, Kadir Yildirim, Kemal Yilmaz, Sercan Yilmaz, Mehmet Yoldas, Ahmet Yuce, Mehmet Ozgur Yucel, Cem Nedim Yuceturk, Jean de la Rosette, Ates Kadioglu

**Affiliations:** 1 Department of Urology, Taksim Education and Research Hospital, Istanbul, Turkey; 2 Department of Urology, Bağcılar Education and Research Hospital, Istanbul, Turkey; 3 Istanbul Provincial Health Directorate, Istanbul, Turkey; 4 Department of Urology, Elazığ City Hospital, Elazığ, Turkey; 5 Department of Urology, Esenyurt Necmi Kadıoğlu State Hospital, Istanbul, Turkey; 6 Department of Urology, Ankara City Hospital, Ankara, Turkey; 7 Department of Urology, Ankara Gülhane Training and Research Hospital, Ankara, Turkey; 8 Department of Urology, Bursa Yüksek Ihtisas Training and Research Hospital, Bursa, Turkey; 9 Department of Urology, Katip Çelebi University, School of Medicine, Izmir, Turkey; 10 Department of Urology, Okmeydanı Education and Research Hospital, Istanbul, Turkey; 11 Department of Urology, Haydarpaşa Education and Research Hospital, Istanbul, Turkey; 12 Department of Urology, Erciyes University, School of Medicine, Kayseri, Turkey; 13 Department of Urology, Gaziantep University, School of Medicine, Gaziantep, Turkey; 14 Department of Urology, Adıyaman University, School of Medicine, Adiyaman, Turkey; 15 Department of Urology, Bozyaka Training and Research Hospital, Izmir, Turkey; 16 Department of Urology, Şişli Etfal Training and Research Hospital, Istanbul, Turkey; 17 Department of Urology, Kartal Dr Lütfi Kırdar Training and Research Hospital, Istanbul, Turkey; 18 Department of Urology, Evliya Çelebi Training and Research Hospital, Kütahya, Turkey; 19 Department of Urology, Göztepe Medeniyet University, School of Medicine, Istanbul, Turkey; 20 Department of Urology, Medipol University, School of Medicine, Istanbul, Turkey; 21 Department of Urology, Biruni University, School of Medicine, Istanbul, Turkey; 22 Department of Urology, Karabük University, School of Medicine, Karabuk, Turkey; 23 Department of Urology, Van YY University, School of Medicine, Van, Turkey; 24 Department of Urology, Aydın University, School of Medicine, Istanbul, Turkey; 25 Department of Urology, Istanbul University, Istanbul School of Medicine, Istanbul, Turkey; 26 Department of Urology, Giresun University, School of Medicine, Giresun, Turkey; 27 Department of Urology, Dışkapı Training and Research Hospital, Ankara, Turkey; 28 Department of Urology, Sadi Konuk Training and Research Hospital, Istanbul, Turkey; 29 Department of Urology, Koru Hospital, Ankara, Turkey; 30 Department of Urology, Cumhuriyet University, School of Medicine, Sivas, Turkey; 31 Department of Urology, Bursa Education and Research hospital, Bursa, Turkey; 32 Department of Urology, Keçiören Training and Research Hospital, Ankara, Turkey; 33 Department of Urology, Ümraniye Training and Research Hospital, Istanbul, Turkey; 34 Department of Urology, Bahceşehir University, School of Medicine, Istanbul, Turkey; 35 Department of Urology, Fırat University, School of Medicine, Elazığ, Turkey; 36 Department of Urology, Adana City Hospital, Adana, Turkey; 37 Department of Urology, Eskişehir City Hospital, Eskişehir, Turkey; 38 Department of Urology, Samsun Training and Research Hospital, Samsun, Turkey; 39 Department of Urology, Balıkesir University, School of Medicine, Balıkesir, Turkey; 40 Department of Urology, Akçakale State Hospital, Şanlıurfa, Turkey; 41 Department of Urology, aziosmanpaşa Training and Research Hospital, Istanbul, Turkey; 42 Department of Urology, Elazığ City Hospital, Elazığ, Turkey; 43 Department of Urology, Gazi University, School of Medicine, Ankara, Turkey; 44 Department of Urology, Kahta State Hospital, Adıyaman, Turkey; 45 Department of Urology, Atatürk University, School of Medicine, Erzurum, Turkey; 46 Department of Urology, Istinye University, School of Medicine, Istanbul, Turkey; 47 Department of Urology, FSM Training and Research Hospital, Istanbul, Turkey; 48 Department of Urology, Şanlıurfa Training and Research Hospital, Şanlıurfa, Turkey; 49 Department of Urology, Rize Training and Research Hospital, Rize, Turkey; 50 Department of Urology, Medical Park Hospital, Elazığ, Turkey; 51 Department of Urology, Malatya Training and Research Hospital, Malatya, Turkey; 52 Department of Urology, Gülhane Training and Research Hospital, Ankara, Turkey; 53 Department of Urology, Tepecik Training and Research Hospital, Izmir, Turkey; 54 Department of Urology, Darende State Hospital, Malatya, Turkey; 55 Department of Urology, Ankara Training and Research Hospital, Ankara, Turkey; 56 Department of Urology, Section of Andrology, Istanbul University, Istanbul School of Medicine, Istanbul, Turkey On Behalf of Turkish Urology Academy

**Keywords:** Priapism, andrology, penile prosthesis

## Abstract

**Objective:**

The aim of this study is to evaluate current urologic practice regarding the management of priapism in Turkey and compare with international guidelines.

**Methods:**

Urologists and urology residents were invited to an online survey consisting of 30 multiple-choice questions on priapism-related clinical practices that were considered most important and relevant to practices by using Google Forms.

**Results:**

Total number of responses was 340. Respondents reported that they recorded a detailed patient’s medical history and physical examination findings (n = 340, 100%) and laboratory testing, which includes corporal blood gas analysis (n = 323, 95%). Participants announced that they performed Doppler ultrasound for 1/4 cases (n = 106, 31%), but 22% of the participants (n = 75) replied that they performed in >75% of cases. Participants (n = 311, 91%) responded that the first-line treatment of ischemic priapism is decompression of the corpus cavernosum. Moreover, most respondents (n = 320, 94%) stated that sympathomimetic injection drugs should be applied as the second step. About three-quarters of respondents (n = 247, 73%) indicated adrenaline as their drug of choice. Phosphodiesterase type 5 inhibitors seems to be the most preferred drug for stuttering priapism (n = 141, 41%). Participants (n = 284, 84%) replied that corpora-glanular shunts should be preferred as the first. A large number of participants (n = 239, 70%) declared that magnetic resonance imaging can be performed in cases with delayed (>24 hours) priapism to diagnose corporal necrosis. Most of the participants (84%) responded that penile prosthesis should be preferred to shunts in cases with delayed (>48 hours) priapism.

**Conclusion:**

It would be appropriate to improve the training offered by professional associations and to give more training time to the management of priapism during residency.

**Main Points**

- This survey provides a snapshot of Turkish urology and their management of Priapism.
- The manuscript evaluates the current practice patterns in the management of priapism among urologists and sheds light on the heterogeneity between physicians and guidelines priapism work-up and management.
- According to the survey results, it appears that some of the diagnostic and therapeutic approaches used by urologists who participate in the survey are at odds with internationally accepted standards.

## Introduction

Priapism is a urologic condition described as a prolonged penile erection of more than 4 hours that persists after or unrelated to sexual activity.^1^ It can be divided into 3 groups: ischemic, non-ischemic, and stuttering priapism. Ischemic or low-flow priapism accounts for 95% of all priapism cases with an estimated incidence rate of 5.3 per 100 000 men per year in the United States.^2^ Ischemic priapism is characterized by a persistent painful erection with little or no cavernous arterial inflow.^3^ It has time-dependent changes on metabolic conditions of corporal smooth muscle with progressive hypercarbia, acidosis, and hypoxia that result in fibrosis and erectile dysfunction (ED).^4,5^ The length of the ischemic episode indicates the most considerable predictor for the development of corporal fibrosis and ED; therefore, ischemic priapism requires prompt diagnosis and treatment.^6^ Aspiration and irrigation with saline of the corpora cavernosa or injection of sympathomimetic agents are strongly recommended as a first-line treatment.^7,8^ Second-line treatment options typically refer to surgical techniques for refractory or delayed ischemic priapism in the form of penile shunt surgery and penile prosthesis implantation (PPI) and should only be considered when other conservative treatment options have failed.^7,8^ Stuttering (recurrent or intermittent) and non-ischemic (high flow) priapism are rarer and could be managed on a non-emergency basis once the diagnosis has been established.

Due to the low incidence rate, literature concerning the emergency management of priapism consists mostly of case reports and studies consisting of a small number of patients. There is only one study from the UK in literature that has evaluated urologists’ approaches to the diagnosis and management of episodes of priapism.^9^ The European Association of Urology (EAU) and the American Urological Association (AUA) have provided guidelines based on established clinical practice and existing published literature.^7,8^ The current challenges in the management of priapism include determining the true timing and benefit of shunt surgeries and PPI. Moreover, the role of treatment options for priapism and timing remains controversial and there is still no consensus as to the management of high flow and stuttering priapism. This study aimed to evaluate current urologic practice patterns regarding the management of priapism and investigate the knowledge and approaches of qualified urologists and residents in Turkey about the diagnosis and treatment of priapism.

## Material and Methods

### Survey Design and Participants

After the approval of the study by the Gaziosmanpaşa Education and Research Hospital ethics committee (2021/22), urologists and residents in Turkey were invited on December 17, 2021, to participate in an online survey consisting of 30 questions relating to the frequency of cases encountered and emergency management steps including diagnosis, first line, and surgical treatments (Supplementary File 1). The written informed consent was obtained from all participants. Data were captured using the online survey application Google Forms.

### Statistical Analysis

The survey responses were downloaded and saved as separate files and duplicate responses were excluded from the analysis. Summary statistics were calculated using Statistical Package for Social Sciences version 22.0 for Windows (IBM SPSS Corp.; Armonk, NY, USA). Each response was reported as a percentage of the number of respondents for that question.

## Results

### Demographics of Survey Participants

The total number of responses received by the end of the survey was 340. The majority of the respondents were aged 30-39 years old (n = 138, 41%), followed by 40-49 years old (n = 86, 25%), 50-59 years old (n = 62, 18%), less than 30 years old (n = 35, 10%), and over 60 years old (n = 19, 5%) (Table 1).

The majority of the respondents were clinically experienced less than 10 years (n = 143, 42%), followed by 10-20 years (n = 114, 34%), and by more than 20 years (n = 83, 24%). The largest number of the participants that responded were qualified urologists (n = 241, 71%), others were residents in urology (n = 62, 18%) or had an academic degree in urology (n = 37, 11%). The respondents mostly work at a university or teaching hospital (n = 237, 70%) and others in state hospitals (n = 53, 16%) or a private hospital/clinic (n = 50, 15%) (Table 1).

The respondents’ frequency of performing priapism treatment was very high (n = 332, 98%); nevertheless, the number who had received special training for the treatment of priapism (n = 39, 11.4%) and had performed treatment more than 5 times in a year (n = 56, 16%) were low (Table 1).

### Method of Diagnosis of Priapism

Almost all of the respondents stated that they knew the clinical definition of ischemic and non-ischemic priapism (n = 326, 96%) and they answered correctly about the etiology of non-ischemic priapism (n = 313, 92%).

**Figure 1.**
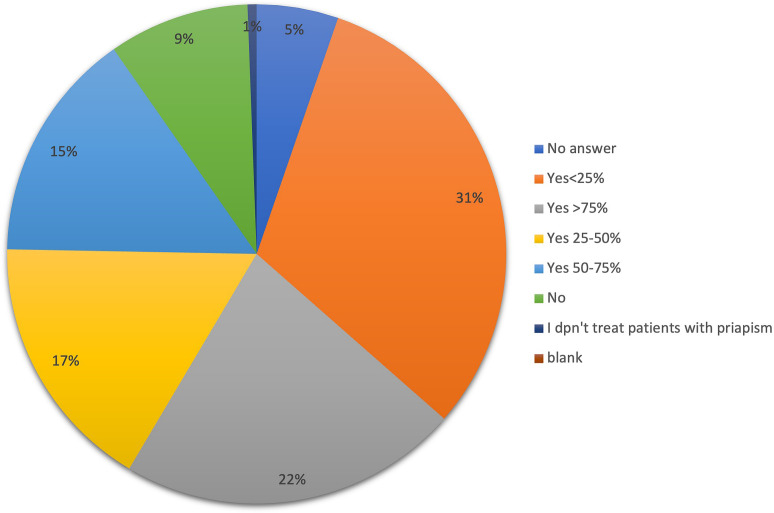
Penile color doppler ultrasound usage rate in the diagnosis of a patient with priapism.

**Figure 2.**
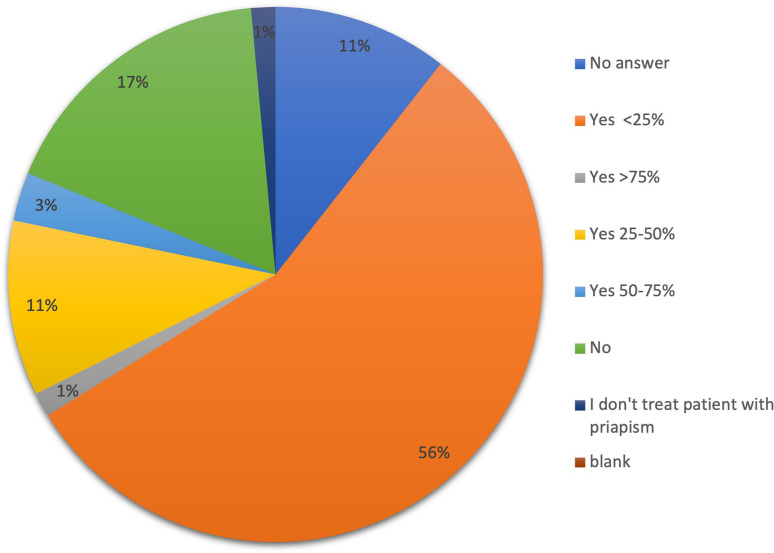
Use of penile magnetic resonance imaging in the evaluation of a patient with priapism.

All participants responded that they take the patient’s medical history and conduct a physical examination of the penis as the first step (n = 340, 100%), and a large number of respondents also noticed that they perform a perineal and abdominal examination (n = 325, 95%). Furthermore, most of the respondents declared that they obtain laboratory testing, which includes a complete blood count, platelet count, white blood cell count, and coagulation profile (n = 334, 98%). Penile blood gas analysis is also a widely performed diagnostic test among the participants (n = 323, 95%). Most of the participants announced that they performed a color Doppler ultrasound of the penis and perineum during a clinical diagnosis for 1 in 4 cases (n = 106, 31%), but 22% of the participants (n = 75) replied that they performed this test in 3 in 4 cases, 17% of the participants use this test in 25%-50% of the cases and 15% in 50%-75% of the cases (Figure 1). Finally, 56% (n = 189) of the participants responded that they performed penile MRI for 1 in every 4 cases, 11% (n = 36) in 25%-50% of cases, and 17% (n = 59) mentioned that they do not use penile MRI for clinical diagnosis (Figure 2).

### Practices of Participants in the Conservative and First-Line Treatment Options of Priapism

**Figure 3.**
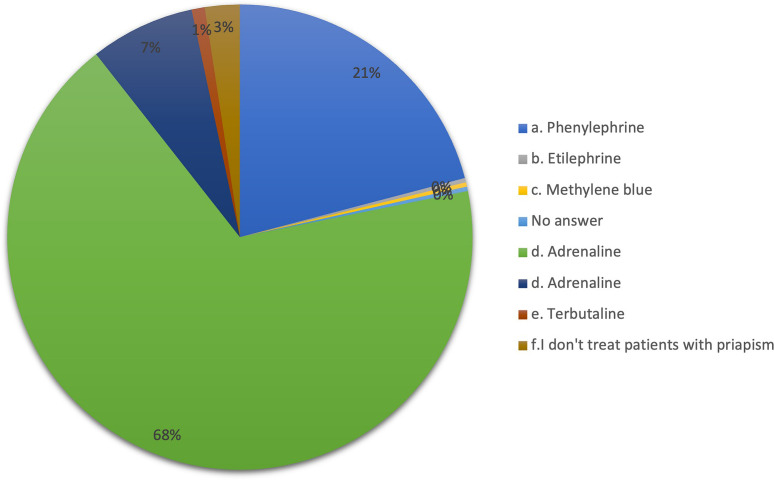
Which medical treatment do you prefer for sympathomimetic drug injection for the medical treatment of ischemic (low-flow) priapism?

A major part of the participants (n = 311, 91%) responded that the treatment of ischemic (low flow) priapism is decompression of the corpus cavernosum with penile aspiration until bright red arterial blood is obtained. Moreover, 94% of respondents (n = 320, 94%) stated that an intracavernous injection of a sympathomimetic drug should be applied as a second step for persistent ischemic (low flow) priapism after the failure of aspiration alone. About three-quarters of the respondents (n = 247, 73%) indicated that adrenaline as their sympathomimetic drug of choice for the first-line medical treatment and phenylephrine as the second most widely chosen agent (n = 78, 23%), whereas terbutaline had a percentage of one (n = 4, 1%) and methylene blue was a choice for only one respondent (Figure 3).

More than half of the respondents noticed that at least 1 hour of aspiration, irrigation, and the administration of sympathomimetic drugs should be applied before surgical options are considered (n = 225, 66%). In addition, 40 participants (n = 40, 11%) said that first-line treatment should be applied once before surgery, and 51 participants (n = 51, 15%) choose to apply first-line treatments for at least 4 hours.

**Figure 4.**
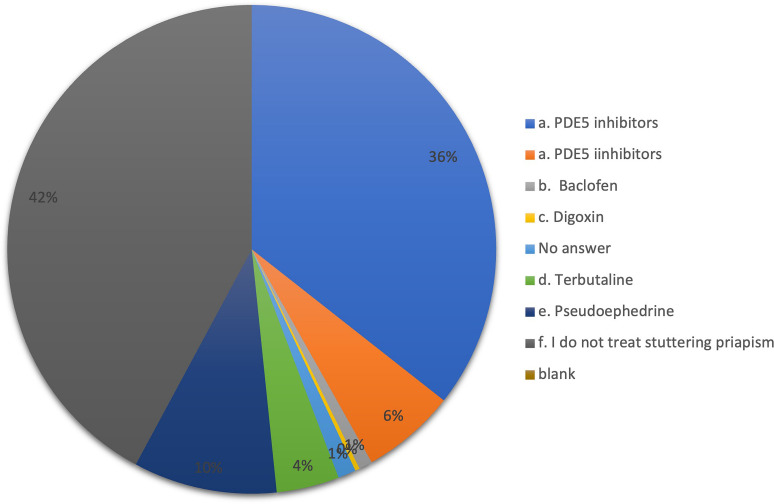
What pharmacological agents do you use for the medical treatment of stuttering priapism?

Most of the respondents suggested that stuttering priapism could cause significant penile fibrosis if left untreated (n = 215, 63%). Although the phosphodiesterase type 5 (PDE-5) inhibitors seem to be the most preferred drug of the participants (n = 141, 41%), a significant portion stated that they do not do medical treatment for stuttering priapism patients primarily (n = 142, 42%). Other agents used in the medical treatment of stuttering priapism in the first instance, according to the participants, were given as pseudoephedrine (n = 35, 10%), terbutaline (n = 14, 4%), and baclofen (n = 3, 1%) (Figure 4).

A significant number of participants mentioned that arterial (high flow) priapism is not a medical emergency (n = 262, 77%), whereas 97% of the participants (n = 329, 97%) responded that ischemic priapism is an emergency and urgent intervention is mandatory. For the management of non-ischemic priapism, respondents mostly declared that conservative management, such as applying ice to the perineum or perineal compression, can be considered (n = 270, 79%).

## Practices of Participants in the Surgical Treatment of Priapism

The major of respondents (n = 330, 97%) mentioned that surgical treatment should be considered for ischemic (low flow) priapism after the failure of aspiration and irrigation with 0.9% saline solution in combination with an intracavernous injection of pharmacological agents. In addition, most of the participants (n = 284, 84%) replied that open distal (corpora-glanular) shunts should be preferred as the first choice for a surgical approach.

A large number of participants (n = 239, 70%) declared that a Gadolinium-enhanced penile MRI can be performed in cases with delayed (>24 hours) priapism to diagnose smooth muscle necrosis. About one-third of the participants (n = 95, 28%) responded that they did not know whether MRI was used to detect smooth muscle necrosis.

Half of the respondents (n = 170, 50%) announced that the administration of acetylsalicylic acid, clopidogrel, and heparin may reduce the recurrence of priapism in perioperative treatment. However, 10% of the respondents replied that there was no additional benefit of this treatment, while 40% emphasized that they did not know whether anticoagulant treatment was used in the first-line or surgical treatment of priapism.

Most of the participants (n = 284, 84%) in the study responded that penile prosthesis surgery should be preferred to proximal or distal shunt techniques in cases with delayed (>48 hours) priapism, and 91% (n = 310) of the participants said that the patient should be immediately consulted for PPI. Almost all of the respondents stated that early PPI preserves penile length and volume, prevents cavernosal fibrosis, reduces infection rates, and lowers penile shortening and revision rates compared to late implantation (n = 325, 96%).

Furthermore, many of the participants (n = 285, 84%) showed a consensus on selective arterial embolization as a recommended treatment method for arterial (high flow) priapism with a high success. On the other hand, the participants declared that arterial ligation of the fistula is possible with a inguinoscrotal or transcorporeal approach and surgery is challenging and carries significant complication risks (n = 288, 85%).

### Posttreatment Complications

A large number of participants (n = 201, 59%) reported in the study that the maintenance of sexual function with arterial (high flow) priapism would be higher than 75%. However, most participants (n = 275, 81%) noticed that the maintenance of sexual functioning in ischemic (low-flow) priapism lasting longer than 36 hours would be less than 25%. About one-fourth of respondents (n = 83, 24%) said that they did not have any knowledge about this information.

## Discussion

Priapism is a rare pathology for a large proportion of urologists in our country, and, according to the survey results, a significant number of urologists have only 1-2 cases per year. This finding is consistent with large-scale observational studies reporting an extremely rare incidence rate (5/100 000).^2^ This survey is the first study to identify the current clinical management of priapism in Turkey.

The majority of the participants emphasized that a complete blood count, coagulation parameters, and cavernous blood gas measurements were performed according to the literature and international guidelines.^7,8^ However, when asked if imaging of the penis with Doppler ultrasound was performed, participants reported different approaches. According to international guidelines and current literature, the role of a Doppler ultrasound in diagnosis is controversial.^7,8^ Although it can detect the location of the fistula with a sensitivity of 100% and a specificity of 73% in cases of high-flow priapism, reactive hyperemia may develop in the proximal corpus cavernosum with the high arterial flow after aspiration, which may lead to an incorrect diagnosis of non-ischemic priapism.^10,12^

Almost all participants reported intracavernosal injection of a sympathomimetic after aspiration and irrigation as the first-line treatment for ischemic priapism. In contrast to phenylephrine, the most commonly used intracavernosal sympathomimetic in the treatment of ischemic priapism worldwide, the survey results showed that epinephrine is the most commonly used drug in Turkey. Both EAU and AUA guidelines recommend phenylephrine (α-1 adrenergic agonist with minimal β-adrenergic effect) as the first-line medical treatment.^7,8^ The corpus cavernosum mainly contains α1a (44%), α2a (34%), and α1b (22%) adrenergic receptors. Phenylephrine should be diluted right before intracavernosal injection to a concentration of 100-500 µg/mL and a solution should be prepared by adding 10 mg/mL phenylephrine to 19 mL physiological saline.^13,14^ Before deciding on the treatment failure, the recommendation is to apply 1 mL of the solution every 3-5 minutes for about 1 hour. Epinephrine, which is more commonly used in Turkey, is administered every 20 minutes, up to 5 times, at a dose of 1/100 000.^15,16^ The reason for the more frequent administration of epinephrine in Turkey can be explained by the absence of phenylephrine.

In the survey, when asked how long first-line treatment should be given for ischemic priapism, about 30% of the participants gave a different answer from the time given in the literature and international guidelines which recommends that it should be given for at least one hour.^7,8^ Although there is no definitive consensus on the duration of first-line treatment, it is known that the most important predictive factor for corporal smooth muscle viability in the treatment of priapism is the duration and that blood flow in the corpora cavernosa should be restored as soon as possible.^17,18^ Interstitial edema, sinusoidal endothelial contraction, and thrombocyte aggregation are observed in the 12-24th hour of ischemic priapism. Thrombosis of the sinusoidal spaces, smooth muscle necrosis, and fibrosis in the 48th hour of ischemic priapism was also observed.^19^

As recommended by international guidelines, survey respondents answered that the first surgical intervention for ischemic priapism for which primary treatment was unsuccessful should be the distal corporo-glandular shunt.^7,8^ Penile shunt surgery aims to create drainage for the ischemic blood from the corpora cavernosa to glans penis, proximal corpus spongiosum, or a vein.^1,20,21^ Although the type of shunt procedure depends on the surgeon’s preference and experience with the technique, distal shunt procedures should be performed before considering a proximal shunt. Distal shunt procedures have been found to be superior to proximal shunt techniques in terms of preserving erectile function.^22^

A penile MRI can show corporal smooth muscle necrosis with high sensitivity (up to 100%).^11^ The EAU guideline recommends that the extent of necrosis should be determined by penile MRI and treatment should be designed accordingly, especially in patients with ischemic priapism between 24 and 48 hours.^7^ Approximately one-third (n = 101) of survey respondents reported that they do not use MRI in the diagnosis and treatment of priapism.

Although AUA does not recommend antithrombotic therapy due to insufficient evidence, the recurrence rate has been reported to be reduced by the administration of heparin or acetylsalicylic acid prior to the procedure in patients given sympathomimetics along with aspiration and irrigation.^8,23^ The use of antithrombotic treatments (325 mg acetylsalicylic acid preoperatively, 5000 IU heparin intraoperatively, clopidogrel 75 mg, and acetylsalicylic acid 81 mg for 5 days postoperatively) in shunt surgery has been reported to have effects on the resolution of priapism and prevention of recurrence. In one study, for a group of patients who had failed first-line treatments and underwent shunt surgery, the number of priapism recurrences decreased by 84% in those who received perioperative antithrombotic therapy compared with the group who did not receive this therapy.^23^ About half of the survey participants reported that they did not know about antithrombotic therapies in the treatment of priapism.

Treatment-resistant acute ischemic priapism (>48 hours) usually results in complete ED (100%) with marked long-term shortening of the penis.^24^ It was also found that >50% of patients with priapism lasting 24-48 hours had permanent ED.^25^ In these cases, early penile prosthesis surgery is recommended. The AUA guidelines recommend PPI in patients with a priapism episode that lasted more than 36 hours.^8^ While EAU guidelines recommend prosthetic surgery for episodes lasting longer than 48 hours, they recommend evaluating the viability of cavernosal smooth muscle with penile MRI between 24 hours and 48 hours.^7^ In the line, 84% of participants stated that they refer their patients for PPI in these late cases. Delayed PPI is not recommended due to the high complication rate and difficulty of the technique. Early PPI is also important to maintain penile length and thickness and prevent the development of fibrosis. Early PPI has a lower rate of infection (6%-7% vs. 19%-30%) and revision (9% vs. 27%) than late PPI.^26^ The optimal time for early PPI is in the first 3 weeks after priapism.^21^ In patients who have undergone shunt surgery, PPI can be deferred until the edema in the penis subsides and the wound heals. During this postponement, vacuum devices can be helpful to prevent fibrosis, penile shortening, and deformity.^25^

Stuttering priapism is defined as the recurrent form of ischemic priapism.^27^ The erections are self-limiting, include intermittent periods of detumescence and the duration of the erection is shorter than in ischemic priapism.^27^ The frequency and duration of episodes are variables.^28^ A single episode may develop into persistent ischemic priapism.^28^ Untreated cases, similar to ischemic priapism, lead to corporeal fibrosis and ED. However, 19% of survey participants said that stuttering priapism would not cause ED, and 18% did not know if it would cause ED. Only half of the participants reported that they start medical treatment for stuttering priapism and the most commonly used medications are PDE-5 inhibitors. According to the literature, there are not enough studies with a high level of evidence on the efficacy and safety of current treatments.^29^ The international guidelines do not provide clear recommendations on the superiority of medical treatments for stuttering priapism.^7,8^

Although 77% of participants did not consider non-ischemic priapism to be a urological emergency, it has been shown that supraphysiological oxygen levels in non-ischemic priapism result in loss of corporal smooth muscle and ED.^30^ Therefore, the treatment of non-ischemic priapism should also be immediate. Non-ischemic priapism usually results from a fistula causing increased arterial flow between the cavernosal artery, or its branches, and the corporal sinusoidal spaces due to pathologies, such as a blunt perineal, penile trauma, or malignancy. Conservative treatments have proven successful, especially when the fistula is located in the branches of the cavernosal artery.^31-33^ Predominance of participants (70%) noticed that the use of the cold application and compression should be the first step in non-ischemic priapism, while 84% declared that selective arterial embolization should be performed in cases where conservative treatments have been unsuccessful.

Despite the interesting findings of the current survey, a few limitations are noted. First, lack of patient outcomes. Patient results were not questioned and not included. Second, since large amounts of data are difficult to tabulate, the results section is a little longer.

Priapism is a urological emergency rarely encountered by most urologists in Turkey. This survey showed a marked diversity of priapisim management and identifies many gaps in the guidelines, thus highlighting a number of areas where further research is needed. This current survey shows that some urologists need to improve their knowledge and procedural skills in some important treatment steps. It would be appropriate to improve the training offered by professional associations and to give more training time to the diagnosis and treatment of priapism during residency. As recommendations from international guidelines may differ, the standardization of priapism treatment can be achieved by developing up-to-date guidelines specifically for Turkey.

## Figures and Tables

**Table 1.** Demographics of Respondents

Age	n	%
25-35 years	106	31
36-50 years	153	45
>50 years	81	24
**Clinical experience**		
0-5 years	64	19
5-10 years	79	23
10-20 years	114	34
>20 years	83	24
**Academic title**		
Resident	62	18
Qualified urologist	241	71
Academician	37	11
**Institution**		
State hospital	53	15
Training/university hospital	237	70
Private clinic	50	15
**Special training for priapism management**		
Yes	39	11
No (only in residency)	301	89

**Table:** 

	Yes <25%	Yes 25-50%	Yes 50-75%	Yes >75%	No	Do not treat priapism
Taking history						
Physical examination of the penis						
Physical examination abdomen and perineum						
Complete blood count						
Coagulation profile						
Blood gas analysis of the corpus cavernosum						
Penile Color duplex ultrasound

## Supplementary File 1.

We invite you to complete the following survey which is prepared by the Turkish Academy of Urology and related to the management of PRIAPISM. The outcome of the survey will be presented during the Urology Chronicles and eventually published in the Turkish Journal of Urology.

1. What is your age? (*Please specify*) ___________________
2. How many years are you in clinical practice?
  - 0- 5/year
  - 5-10/year
  - 11-15/year
  - 16-20/year
  - > 20/year
3. Which one of the following best describes the stage of your career?
  - I’m a qualified urologist
  - I’m a resident in training
  - I’m enrolled in a fellowship
  - Other (*please specify*) ___________________
4. Which one of the following best describes your practice setting?
  - Academic/University hospital
  - Public non-academic hospital
  - Private practice (office and/or hospital)
5. Do you treat patients for Priapism?
  - Yes
  - No
6. Did you follow specific training for treatment of priapism? (Please indicate any that apply)
  - During my residency yes/no
  - During a (short) fellowship yes/no
  - I followed in-person instructional courses yes/no
  - I followed online instructional courses yes/no
  - I have been reading about specific treatments yes/no
  - I have watched surgical recordings on demand yes/no
  - No, I did not receive any specific training yes/no
7. How many patients with priapism do you treat annually?
  - No patients
  - 1-2/year
  - 3-5/year
  - >5/year
8. I perform the following investigations before treating a patient with priapism

Are you familiar with the difference between ischemic (low flow) priapism and arterial (high flow) priapism?
  - yes
  - no
The initial management of ischemic (low flow) priapism includes decompression of the corpora cavernosa by penile aspiration until fresh red blood is obtained
  - Yes
  - No
  - I do not know
In ischemic (low flow) priapism that persists despite aspiration, the next step is intracavernous injection of a sympathomimetic drug
  - Yes
  - No
  - I do not know
Which medical treatment for ischemic (low flow) priapism do you use:
  - Phenylephrine yes/no
  - Etilephrine yes/no
  - Methylene blue yes/no
  - Adrenaline yes/no
  - Terbutaline yes/no
How long should aspiration and sympathomimetic injection be performed before moving on to surgical treatment?
  - Should only be performed once
  - Should be tried for at least an hour
  - Should be tried for at least four hour
  - I do not know
Surgical treatment is recommended in ischemic (low flow) priapism when blood aspiration and intracavernous injection of sympathomimetic drugs have failed.
  - Yes
  - No
  - I do not know
Distal shunt surgical procedure should be preferred first
  - Yes
  - No
  - I do not know
Gadolinium-enhanced MRI may be useful to diagnose smooth muscle necrosis in cases of delayed or refractory priapism (>24 hours).
  - Yes
  - No
  - I do not know
Peri- and post-operative anticoagulation with ASA, clopidogrel and heparine in surgical treatment of ischemic priapism may decrease priapism recurrence.
  - Yes
  - No
  - I do not know
A penile prosthesis should be preferred over proximal and distal shunting particularly in delayed and refractory priapism (> 48 hours).
  - Yes
  - No
  - I do not know
In cases of ischemic (low flow) priapism presenting 48 hours after onset, or in cases for which all interventions have failed, erectile dysfunction is inevitable, and the immediate implantation of a penile prosthesis should be discussed with the patient
  - Yes
  - No
  - I do not know
Early PPI offers the opportunity to maintain penile length, girth, prevent cavernosal fibrosis and associated with lower infection rates, penile shortening and revision rates compared to late insertion.
  - Yes
  - No
  - I do not know
Stuttering priapism is similar to ischaemic priapism in that it is low-flow and ischaemic and, if left untreated, can result in significant penile fibrosis.
  - Yes
  - No
  - I do not know
Which pharmacological agent for stuttering priapism do you use?
  - 5PDEi yes/no
  - Baclofen yes/no
  - Digoxin yes/no
  - Terbutaline yes/no
  - Pseudoephedrine yes/no
Arterial (high flow) priapism usually occurs after blunt perineal or penile trauma
  - Yes
  - No
  - I do not know
The management of arterial (high flow) priapism is not an emergency
  - Yes
  - No
  - I do not know
The management of ischemic (low flow) priapism is not an emergency
  - Yes
  - No
  - I do not know
Conservative management of arterial (high flow) priapism includes the use of ice applied to the perineum or site-specific perineal compression.
  - Yes
  - No
  - I do not know
Selective artery embolization is the suggested treatment modality for arterial (high flow) priapism and has high success rates
  - Yes
  - No
  - I do not know
Surgical ligation of the fistula is possible through a transcorporeal or inguinoscrotal approach in arterial priapism but surgery is technically challenging and associated with significant risks, particularly of ED.
  - Yes
  - No
  - I do not know
The preservation rate of sexual function in arterial (high flow) priapism is
  - <25%
  - 25-50%
  - >75%
  - I do not know
The preservation rate of sexual function in ischemic (low flow) priapism >36 hours is
  - <25%
  - 25-50%
  - >75%
  - I do not know

We sincerely thank you for your collaboration.
